# Diagnostic Stewardship in Lower Respiratory Tract Infections Using Procalcitonin

**DOI:** 10.1017/ash.2021.58

**Published:** 2021-07-29

**Authors:** Amanda Gusovsky, David Burgess, Donna Burgess, Emily Slade, Chris Delcher, Alison Woodworth, Jordan Chael, Thai Osborne

## Abstract

**Background:** A team of infectious diseases physicians, infectious diseases pharmacists, clinical laboratorians, and researchers collaborated to assess the management of lower respiratory tract infections (LRTIs). In 1 sample from our institution, 96.1% of pneumonia cases were prescribed antibiotics, compared to 85.0% in a comparison group. A collaborative effort led to the development of a protocol for procalcitonin (PCT)-guided antibiotic prescribing that was approved by several hospital committees, including the Antimicrobial Stewardship Committee and the Healthcare Pharmacy & Therapeutics Committee in December 2020. The aim of this analysis was to develop baseline information on PCT ordering and antibiotic prescribing patterns in LRTIs. **Methods:** We evaluated all adult inpatients (March–September 2019 and 2020) with a primary diagnosis of LRTI who received at least 1 antibiotic. Two cohorts were established to observe any potential differences in the 2 most recent years prior to adoption of the PCT protocol. Data (eg, demographics, specific diagnosis, length of stay, antimicrobial therapy and duration, PCT labs, etc) were obtained from the UK Center for Clinical and Translational Science, and the study was approved by the local IRB. The primary outcome of interest was antibiotic duration; secondary outcomes of interest were PCT orders, discharge antibiotic prescription, and inpatient length of stay. **Results:** In total, 432 patients (277 in 2019 and 155 in 2020) were included in this analysis. The average patient age was 61.2 years (SD, ±13.7); 47.7% were female; and 86.1% were white. Most patients were primarily diagnosed with pneumonia (58.8%), followed by COPD with complication (40.5%). In-hospital mortality was 3.5%. The minority of patients had any orders for PCT (29.2%); among them, most had only 1 PCT level measured (84.1%). The median length of hospital stay was 4 days (IQR, 2–6), and the median duration of antibiotic therapy was 4 days (IQR, 3–6). **Conclusions:** The utilization of PCT in LRTIs occurs in the minority of patient cases at our institution and mostly as a single measurement. The development and implementation of a PCT-guided therapy could help optimize antibiotic usage in patients with LRTIs.

**Funding:** No

**Disclosures:** None

